# Transcutaneous bilirubin in newborns before, during, and after home phototherapy—Results from a secondary analysis of a randomized controlled trial

**DOI:** 10.1371/journal.pone.0320067

**Published:** 2025-03-25

**Authors:** Felicia Erlandsson Speychal, Miriam Pettersson, Mats Eriksson, Andreas Odlind, Andreas Ohlin

**Affiliations:** 1 Department of Pediatrics, Faculty of Medicine and Health, Örebro University Hospital, Örebro, Sweden; 2 Faculty of Medicine and Health, School of Medical Sciences, Örebro University, Örebro, Sweden; 3 Faculty of Medicine and Health, School of Health Sciences, Örebro University, Örebro, Sweden; 4 Department of Pediatrics, Faculty of Medicine and Health, Falu Hospital, Örebro, Sweden; Bangabandhu Sheikh Mujib Medical University (BSMMU), BANGLADESH

## Abstract

Home phototherapy is recommended as an alternative to hospital-based therapy for neonatal jaundice in otherwise healthy full-term infants. With a reliable device for transcutaneous bilirubin (TcB) measurement, bilirubin values could be monitored at home during treatment. This study aimed to examine the accuracy of TcB measurement of bilirubin levels before, during, and after home phototherapy. Patients requiring phototherapy were assigned to home (intervention) or hospital-based phototherapy (control). Transcutaneous bilirubin measurement was made at the sternum (uncovered skin) and at sacrum (covered by the diaper during treatment). Simultaneously, total serum bilirubin (TSB) level was collected through a blood sample. The agreement between TcB and TSB before, during, and after phototherapy was assessed using Bland-Altman plots. Altogether 141 patients and 856 paired bilirubin values were included. The results show that TcB measurements underestimate TSB levels. Before phototherapy, the mean difference between TcB and TSB was 75 ± 36 μmol/L at the sternum and 135 ± 39 μmol/L at sacrum, with no difference between study groups. During phototherapy, the mean difference at the sternum was larger in the control group, 105 ± 73 μmol/L, than in the intervention group, 50 ± 41 μmol/L; at sacrum, the mean difference was 125 ± 44 μmol/L, comparable in both study groups. After phototherapy, the TcB–TSB agreement improved, with a mean difference of 29 ± 33 μmol/L (sternum) and 87 ± 35 μmol/L (sacrum), and no difference between study groups. In conclusion this study shows that full-term infants who qualified for phototherapy show poor agreement between TcB measurement and TSB, suggesting that TcB measurements cannot replace measurement of TSB level before, during, or after home phototherapy.

## Introduction

Neonatal jaundice is a common condition and 2–5% of all newborns require treatment with phototherapy [1–3]. The purpose of phototherapy is to decrease bilirubin levels, and thus prevent neurotoxic complications [4]. Home phototherapy is a safe and cost-effective alternative to inpatient phototherapy for otherwise healthy full-term infants [5,6]. A previous study has suggested that home phototherapy improves bonding and reduces parental stress [7].

The threshold for phototherapy is determined by levels of total serum bilirubin (TSB) [1,4]. Obtaining TSB requires a blood sample, which is painful for infants [8]. Transcutaneous bilirubin (TcB) measurement is a non-invasive method that provides an instant bedside result. It can be used to estimate TSB levels and is recommended for screening infants to identify those who need further bilirubin sampling [9,10]. If TcB is > 250 μmol/L or within 50 μmol/L of phototherapy threshold, it is recommended to confirm the result by measuring TSB [1,4].

Recent studies show conflicting findings about the accuracy of TcB measurements with higher TSB values [4,11–16]. The agreement between TcB and TSB is impacted by phototherapy as it bleaches the skin [17], although the bleaching process is slower in areas covered from light exposure during treatment [17]. Because of the suggested inaccuracy of TcB with high bilirubin values and the influence of phototherapy on the skin, using TcB measurements for newborns requiring phototherapy when high TSB values are expected presents challenges. A recent systematic review and meta-analysis presented good agreement between TSB and TcB when measured on covered skin before and during phototherapy, but using TcB measurement during phototherapy is not recommended [18]. Further studies are still needed to evaluate the potential utility of TcB after phototherapy has been discontinued [18].

While home phototherapy is recommended as an alternative to hospital-based treatment [4], for logistical reasons it can be difficult to monitor bilirubin levels at home. Either the family needs to return to the hospital with their newborn, or a nurse needs to visit the family at home for blood sampling. However, with a reliable mobile device, bilirubin monitoring could be accomplished at home, which would benefit both the family and the caregiver by improving the quality of care. Studies of TcB measurement during home phototherapy are very limited and only one article was found. In 2008, Reyes et al evaluated the BiliChek device during home phototherapy and concluded that because of insufficient accuracy it could not be recommended [19].

This study aims to determine the reliability of TcB measurement before, during, and after home phototherapy. Additionally, because of lack of recent data on the performance of TcB measurements ≥ 250 µmol/L, the study aimed to investigate the accuracy of TcB measurements for high serum bilirubin levels.

## Materials and methods

### Patients and study protocol

This study was part of a randomized controlled, multicenter trial performed at six hospitals in Sweden [5,6]. For this study, data from three hospitals was used: Örebro University Hospital and two regional hospitals, Central Hospital Karlstad and Falu Hospital. Three hospitals with six included patients were excluded because of missing data. The included infants were enrolled between August 11^th^ 2016 and September 26^th^ 2019. Informed, written consent was obtained from all parents. The study was approved by the Regional Ethical Review Board in Uppsala, diary number 2015/336.

The inclusion criteria were a minimum age of 48 hours, a gestational age > 36 + 0 weeks, and a requirement for phototherapy according to Swedish national guidelines for hyperbilirubinemia [1], namely, a TSB level > 300 μmol/L between 48 and 72 hours age or > 350 μmol/L after 72 hours age. The exclusion criteria were a positive direct antiglobulin test (as an indication of blood group incompatibility), TSB > 400 μmol/L, infection, weight loss > 10%, or any other severe illness. The parents had to be able to speak Swedish, be able to perform phototherapy at home, and agree to return to the hospital for daily checkups.

After inclusion, patients were randomized to home phototherapy (intervention group) or hospital phototherapy (control group). Allocation was made by the responsible nurse or physician by opening a sealed envelope, marked with the study identification number, in consecutive order. Randomization was conducted blockwise, with ten patients per block, using a random sequence generator.

### Measurement of bilirubin

All bilirubin measurements were carried out by the responsible nurse. Transcutaneous bilirubin was measured at the sternum, uncovered during treatment, and at sacrum, covered by a diaper during treatment. Transcutaneous bilirubin measurements were performed using a Dräger Jaundice Meter 105 (JM-105; Dräger, Lübeck, Germany). The mean of three consecutive measurements was calculated according to the manufacturer’s instructions. Simultaneously, TSB was obtained through a blood sample. In Örebro, the blood samples were analyzed at the neonatal unit using the blood gas analyzer GEM500 (Werfen, Barcelona, Spain), or at the local laboratory using a Siemens ADVIA system (Siemens Healthineers, Erlangen, Germany). At the other hospitals, blood samples were analyzed using laboratory methods, in Karlstad with a VITROS 4600 (Ortho Clinical Diagnostics, Raritan, NJ, USA) and in Falun with a Siemens Atellica (Siemens Healthineers, Erlangen, Germany).

Bilirubin measurements were performed several times during treatment as and when requested by the attending physician. Patients in the intervention group returned to the hospital daily for checkups. To compare bilirubin measurements between the study groups, three time points were considered for analysis. The first was before phototherapy, at inclusion into the study. The second was the first measurement during phototherapy. The third was after phototherapy, at the last checkup before the patient was discharged from the phototherapy treatment plan, usually when serum bilirubin decreased spontaneously. All other measurements between time points two and three were considered to have unknown phototherapy exposure since it was unclear from the records whether these samples were collected during or after phototherapy. For analyzing high bilirubin values, paired samples from all time points where TSB was ≥ 250 μmol/L were included.

### Phototherapy

Phototherapy was started as soon as possible after inclusion into the study. The equipment used in the intervention group was a BiliSoft phototherapy system (GE Healthcare, Chicago, IL, USA) using spectral irradiance 35 ± 5 µ W/cm²/nm. Patients in the control group used overhead devices (the most used lamp was a Medela phototherapy lamp (Medela, Baar, Switzerland), with spectral irradiance at 30 µ W/cm²/nm), alternatively to or in combination with BiliSoft. Patients were treated with continuous phototherapy wearing only a diaper and eye mask.

In both groups, a form was filled out by the parents or the responsible nurse recording the duration of phototherapy and any interruptions of > 15 minutes. This data was used to determine the time point when phototherapy was discontinued. In cases where the form was missing information, completing data was found by reviewing the patient’s computerized records.

### Statistics

Data from paper forms was recorded in Microsoft Excel (Microsoft Corp., Redmond, WA, USA). In this article, descriptive data is presented as means and standard deviation (SD). For tests of statistical significance, Student’s *t*-test, chi-square test, or Fisher’s exact test were used depending on data properties. For analyses comparing TSB and TcB, correlations were made using Pearson’s correlation coefficient, and the mean difference ( ± SD) was calculated. The agreement between TSB and TcB was assessed with Bland-Altman plots. The limit of agreement was calculated by multiplying the mean difference ( ± SD) by 1.96. Data from before, during, and after phototherapy, as well as for the intervention and control groups, was analyzed separately. A subgroup analysis was performed including only paired values with TSB ≥ 250 μmol/L. All statistical analyses were conducted using SPSS version 29 (IBM Corp., Armonk, NY, USA). Results were considered statically significant if the p-value was < 0.05.

## Results

Altogether 141 infants were included in the study, 65 in the control group and 76 in the intervention group. Of these, 92 (65%) were included at Örebro University Hospital, 32 (23%) at Central Hospital Karlstad, and 17 (12%) at Falu Hospital. The background characteristics of the included patients are presented in Table 1. Three patients in the intervention group were readmitted to the hospital because of high bilirubin values at follow-up, they all responded well to intensified phototherapy. No patient needed an exchange transfusion. Three patients interrupted home phototherapy at their parents’ request; they received standard care at the hospital. Three patients in the intervention group received hospital treatment, which was a protocol violation. All patient data were analyzed in the group to which they were originally randomized, according to the intention-to-treat principle.

**Table 1:** Background characteristics of the included patients.

	All patients n = 141	Intervention n = 76	Control n = 65	p-value
Sex (male/female), n	92/49	43/33	49/16	<0.001
Gestational age (wks + d), mean (SD)	39 + 0 (1 + 3)	39 + 2 (1 + 3)	38 + 5 (1 + 3)	0.046
Delivery (V/C/I), n	111/7/23	62/2/12	45/5/11	<0.001
Birth weight (g), mean (SD)	3,585 (546)	3,596 (539)	3,572 (559)	0.794
Age at inclusion, d, mean (SD)	4.1 (1.1)	4.1 (1.2)	4.1 (1.0)	0.823
Hemoglobin at inclusion, g/L, mean (SD)	192 (19)	196 (17)	188 (21)	0.014
Length of phototherapy, hrs, mean (SD)	22 (12)	21 (11)	23 (13)	0.461

C, Cesarean section; I, instrumental; V, vaginal.

**Fig 1:**
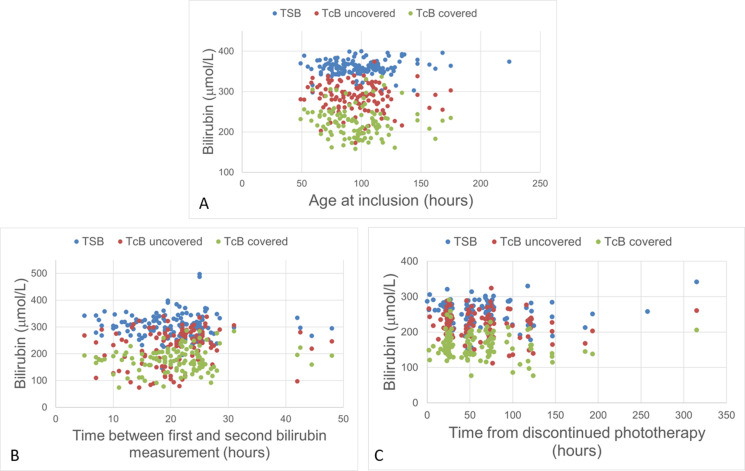
Scatterplot showing total serum bilirubin (TSB) and transcutaneous bilirubin (TcB) measurements. The x-axis shows time in hours; the y-axis gives bilirubin in μmol/L. The sternum is the site for uncovered (exposed) skin and the sacrum for covered skin. A: Before phototherapy. B: During phototherapy. C: After phototherapy.

A total of 856 paired bilirubin measurements were collected, 720 from the three specified time points before, during, and after phototherapy (Fig 1). The number of measurements at each site and time point is given in Table 2. Out of all measurements from the three time points, 587 (82%) were paired measurements with a TSB of ≥ 250 μmol/L. Additionally, 136 paired measurements with a TSB ≥ 250 μmol/L, with unknown exposure to phototherapy, were collected.

**Table 2:** Total serum bilirubin (TSB) and transcutaneous bilirubin (TcB) measurements at the sacrum (covered skin) and sternum (uncovered skin) before, during, and after phototherapy.

	All patients	Intervention	Control	p-value
**Before phototherapy, at inclusion**				
TSB/TcB sternum/TcB sacrum, n	141/116/112	76/62/61	65/54/51	
TSB, μmol/L, mean ± SD	360 ± 18	359 ± 18	361 ± 19	0.581
TcB sternum, μmol/L, mean ± SD	286 ± 34	288 ± 32	283 ± 36	0.373
TcB sacrum, μmol/L, mean ± SD	226 ± 38	224 ± 38	228 ± 37	0.559
**During phototherapy**				
TSB/TcB sternum/TcB sacrum, n	140/124/124	76/71/71	64/53/53	
TSB, μmol/L, mean ± SD	302 ± 47	307 ± 51	296 ± 41	0.142
TcB sternum, μmol/L, mean ± SD	230 ± 67	258 ± 42	193 ± 75	<0.001
TcB sacrum, μmol/L, mean ± SD	178 ± 43	186 ± 47	168 ± 36	0.017
Time after inclusion, hrs, min–max (median)	5–48 (21)	10–48 (24)	5–45 (17)	<0.001
**After phototherapy**				
TSB/TcB sternum/TcB sacrum, n	133/125/119	71/69/68	62/56/51	
TSB, μmol/L, mean ± SD	255 ± 35	252 ± 33	257 ± 36	0.420
TcB sternum, μmol/L, mean ± SD	222 ± 36	221 ± 36	224 ± 36	0.645
TcB sacrum, μmol/L, mean ± SD	164 ± 36	161 ± 37	169 ± 33	0.219
Time after phototherapy, hrs, min–max (median)	0–315 (50)	2–258 (52)	0–315 (47)	0.299

SD, standard deviation.

The mean TSB and TcB levels are presented in Table 2. There were no statistical differences in TSB or TcB level between the study groups, except for TcB during phototherapy administered with skin uncovered, where the mean was 258 μmol/L and 193 μmol/L in the intervention and control group, respectively (p < 0.001). There was a statistical significance between the groups in time from inclusion to the second bilirubin measurement, with a median time of 24 hours in the intervention group and 17 hours in the control group (p < 0.001). There was no statistical difference between groups in how long after discontinued phototherapy the third bilirubin measurement was performed.

The correlation between TSB and TcB before phototherapy was weak and non-significant at both sites. Pearson’s correlation coefficient was r = 0.06 (p = 0.505) for uncovered skin and r = 0.11 (p = 0.251) for covered skin. The correlation strengthened during phototherapy, r = 0.44 (p < 0.001) with the skin uncovered and r = 0.53 (p < 0.001) with it covered. After phototherapy, the strongest correlation was found, r = 0.53 (p < 0.001) and r = 0.51 (p < 0.001) with the skin uncovered and covered, respectively.

Table 3 shows the mean difference between TSB and TcB before, during, and after phototherapy on covered and uncovered skin. Data from both study groups shows that, before phototherapy, TcB measurements on uncovered skin underestimated TSB levels by a mean ( ± SD) of 75 ± 36 μmol/L, and on covered skin by 135 ± 39 μmol/L. In 78% of the TcB measurements at uncovered skin sites and 97% of measurements with covered skin, the underestimate was > 50 μmol/L. The underestimation was similar in both study groups. During phototherapy, the agreement between TSB and TcB values remained similar to that before phototherapy, the mean difference being 74 ± 63 μmol/L and 125 ± 44 μmol/L for uncovered and covered skin, respectively. After phototherapy, there was an improvement in agreement, but measurement of TcB still underestimated TSB level by a mean of 29 ± 33 μmol/L at the sternum (uncovered) and 87 ± 35 μmol/L at sacrum (covered). Except for the measurement at the sternum during phototherapy, where TcB measurement underestimated TSB by a mean of 105 ± 73 μmol/L in the control group, compared with 50 ± 41 μmol/L in the intervention group, no variation in mean difference was observed between the study groups during or after phototherapy. When including all TSB values above the phototherapy threshold with a TSB ≥ 350 μmol/L (patients older than 72 hours), the corresponding TcB at the sternum was ≥ 250 μmol/L in 86% and < 250 μmol/L in 14% of cases.

**Table 3:** Mean difference between total serum bilirubin (TSB) and transcutaneous bilirubin (TcB) levels before, during, and after phototherapy on covered and uncovered skin in newborns treated with home phototherapy versus newborns receiving hospital-based phototherapy.

	Before phototherapy		During phototherapy		After phototherapy	
	Uncovered	Covered	Uncovered	Covered	Uncovered	Covered
**All patients**						
Mean difference (TSB–TcB) ± SD, μmol/L	75 ± 36	135 ± 39	74 ± 63	125 ± 44	29 ± 33	87 ± 35
**Control group**						
Mean difference (TSB–TcB) ± SD, μmol/L	77 ± 37	132 ± 37	105 ± 73	128 ± 42	34 ± 33	87 ± 31
**Intervention group**						
Mean difference (TSB–TcB) ± SD, μmol/L	73 ± 36	137 ± 40	50 ± 41	122 ± 46	26 ± 32	87 ± 38

Data is presented as mean difference ±  standard deviation, SD.

In Supplementary S1 Data, Bland-Altman plots present the agreement between TSB and TcB at covered and uncovered skin sites, before, during, and after phototherapy, for all patients and for the control and intervention group, respectively.

**Fig 2:**
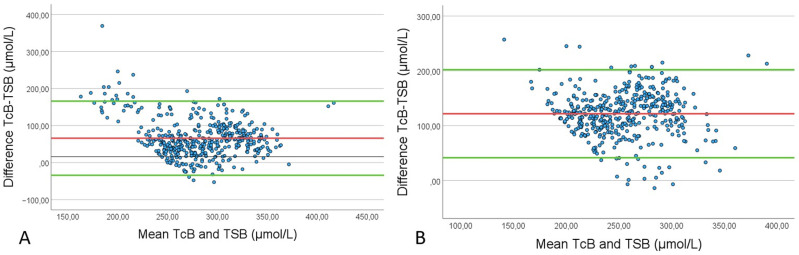
Bland-Altman plots of all paired total serum bilirubin (TSB) and transcutaneous bilirubin (TcB) measurements where TSB ≥ 250 μmol/L, from all time points, regardless of phototherapy exposure. The x-axis shows mean TSB and TcB (μmol/L); the y-axis gives the difference between TSB and TcB (μmol/L). A: shows the agreement between TSB and TcB at the sternum, which was uncovered during treatment. B: presents the agreement between TSB and TcB at sacrum, which was covered by the diaper during treatment.

An additional analysis was conducted, which included all paired samples with a TSB level of ≥ 250 μmol/L from both the control and the intervention group, see Table 4. The samples were divided into four groups based on the exposure to phototherapy. The results showed a decreased correlation between TSB and TcB at uncovered (r = 0.27) and covered (r = 0.23) skin in this subgroup after phototherapy, compared with the correlation observed when including all bilirubin values (r = 0.53 and r = 0.51 with the skin uncovered and covered, respectively). During and before phototherapy, the correlation within the subgroup was similar to that found for all patients. However, the agreement was weaker in this subgroup for both uncovered and covered skin at all time points. The largest increase in mean difference was observed after phototherapy on both uncovered and covered skin. At the sternum, it increased from 29 ± 33 μmol/L to 40 ± 30 μmol/L, and at sacrum, it increased from 87 ± 35 μmol/L to 100 ± 34 μmol/L. The agreement, presented in a Bland-Altman plot for paired values with TSB ≥ 250 μmol/L, is shown in Fig 2.

**Table 4:** Bilirubin levels, the mean difference between total serum bilirubin (TSB) and transcutaneous bilirubin (TcB), and the correlation coefficient when including all paired measurements where TSB ≥ 250 μmol/L.

	TSB	Uncovered skin (sternum)		Covered skin (sacrum)	
	Mean ± SD, µmol/L	MD ± SD, µmol/L	Correlation, *r*	MD ± SD, µmol/L	Correlation, *r*
Before PT (n = 120)	360 ± 22	76 ± 48	0.013	134 ± 41	0.114
During PT (n = 111)	311 ± 42	78 ± 63	0.418	130 ± 40	0.552
After PT (n = 76)	276 ± 18	40 ± 30	0.270	100 ± 34	0.226
Unknown PT (n = 142)	304 ± 33	61 ± 47	0.418	117 ± 40	0.404
Total (n = 449)	316 ± 42	66 ± 51	0.444	122 ± 41	0.536

MD, mean difference; PT, phototherapy; SD, standard deviation.

## Discussion

Home phototherapy is recommended in the American Academy of Pediatrics (AAP) guidelines as an alternative to hospital-based treatment [4]. It may, however, increase the need for a point-of-care testing device. A reliable TcB meter could enable a home care team to monitor the bilirubin values at home during phototherapy, obviating the need for frequent hospital visits by the family and newborn. Therefore, it is of great convenience to assess TcB levels during home phototherapy. This study presents data on the relationship between TSB and TcB before, during, and after phototherapy on covered and uncovered skin, in a population of infants receiving home phototherapy, compared with a control group treated at the hospital.

During phototherapy, a statistically significant difference in TcB on the sternum was observed between the study groups, even though there was no statistical difference in TSB. The TcB on the sternum was lower in the control group. Consequently, the mean difference between TSB and TcB on sternum during phototherapy was higher in the control group compared to the intervention group. While there is no confirmed explanation for this finding, we propose two possible reasons. First, the methods of phototherapy differed between groups. Infants in the hospital received treatment using an overhead device, either alone or in combination with a BiliSoft, while those treated at home only used the BiliSoft. These different methods may have varying skin-bleaching effects. Second, the timing of bilirubin measurement may have contributed to the difference. In the intervention group, there was likely a longer duration between the cessation of phototherapy and bilirubin measurement, as this included travel time to the hospital. As a result, a slight increase in TcB levels may have occurred before the measurements were made in the intervention group. In contrast, the hospital-treated infants could have their bilirubin levels measured almost immediately after discontinued treatment.

The results of our study show that TcB measurements underestimate TSB levels both before and during, as well as after phototherapy. As per AAP guidelines, the recommended safety margin is 50 μmol/L [4], suggesting that TcB values within this range of the phototherapy threshold should be confirmed by measuring TSB. It is also suggested that TcB > 250 μmol/L should be confirmed with TSB [4]. The underestimation in our results was > 50 μmol/L at both sites before and during phototherapy in most cases, and 14% of infants with a TSB above the treatment threshold presented a TcB < 250 μmol/L. This indicates that, according to our data, several infants may have a TSB that exceeds the treatment threshold, even when the recommended decision rule is followed. It is important to note that our data comes from a cohort for which making treatment decisions based on TcB is not recommended. In our study, all patients had a TSB above the treatment threshold before phototherapy and TcB values during phototherapy were performed on skin that had been exposed to light during treatment. Based on the data presented in this study, TcB does not reduce the necessity for blood sampling for TSB before or during phototherapy.

After phototherapy, the agreement was improved, likely due to lower TSB values, as a larger disparity between TSB and TcB values is suggested to be present with higher TSB values [4,11–16]. The mean difference after phototherapy on exposed skin was within the 50 μmol/L limit. However, when assessing the agreement between TSB and TcB, it is important to consider not only the mean difference between the two values but also the limit of agreement. Our data shows a considerable disparity between TSB and TcB, which can exceed the suggested limit in some patients. Although the mean difference was within the suggested safety margin, a considerable number of patients had a corresponding TcB to TSB outside the 50 mmol/L safety margin when considering the standard deviation. Since measurement of TcB tends to underestimate TSB, relying solely on TcB measurements can result in missing high TSB values, which can be harmful to the patient if it means that no treatment is given as a consequence. Furthermore, the difference between TcB and TSB can vary over time and individuals, making it impossible to predict how much a single TcB value will differ from the corresponding TSB.

Previous studies have suggested that TcB measurements are more accurate on covered skin than on exposed skin [18,20], and for this study, it was of interest to evaluate if this was true also during home phototherapy. When infants are treated at home, there is less monitoring of the phototherapy process. For example, if a photo-opaque patch is applied, there is uncertainty as to whether it has been applied at all times. Therefore, in this study, the sacrum was the chosen site for covered skin, as it is naturally protected from light by the diaper. A few previous studies have assessed the accuracy of TcB levels measured at the site covered by a diaper [12,21–25]. Consistent with our findings, other studies also stated that TcB measured at this site underestimates TSB [12,21–25]. After phototherapy, we found a mean difference of 87 ± 35 μmol/L, compared with a mean difference of between 2 μmol/L and 63 μmol/L reported in other studies [12,21–24]. Our study is the first to present data on TcB at sacrum measured during phototherapy in full-terms. Compared with data on preterms, our results show a higher mean difference between TcB and TSB measured under the diaper during phototherapy [22,25]. The underestimation in our results was larger at sacrum than at the sternum. This is in line with the physiological cephalocaudal progression of jaundice [26], meaning that when TSB rises the yellow color is first visible on the head and later in the caudal parts of the body. When designing the study, the availability of a site naturally protected from light exposure during phototherapy was considered favorable, even though lower bilirubin levels at the sacrum were expected because of the cephalocaudal progression of jaundice. The intention was to mathematically adjust for the cephalocaudal progression, but further analyses were not conducted due to poor correlation and agreement with TSB.

Previous studies have suggested that TcB measurement underestimates TSB, with increasing discrepancy at higher TSB values [4,11–16]; we therefore conducted a subgroup analysis on paired bilirubin measurements when TSB was ≥ 250 μmol/L. In line with previous research [4,11–16], our measurements, conducted using the JM-105, showed a greater discrepancy between TcB and TSB at higher TSB values. Our results suggest that this discrepancy is maintained when presenting a high TSB value after discontinued phototherapy. This is important and clinically relevant as patients in need of phototherapy are expected to have high bilirubin levels. Our results contribute to the knowledge that TcB is not a reliable indicator in this group.

Our study has several limitations. Different methods were used to measure TSB, which, in previous research, has been concluded to affect the TSB results [27,28]. In this study, both a blood gas analyzer and different laboratory methods were used, depending on the availability at the different hospitals. Since we did not collect data on the method used for each patient, the possible variability between methods was not considered when comparing TSB with TcB. Also, we did not have complete data on the timing of phototherapy for all patients. This meant that our study population also included a group with unknown phototherapy exposure, where it was not clear if bilirubin measurement had been done during a subsequent phototherapy session following rebound hyperbilirubinemia, or after this repeat treatment. Additionally, the exact time point of the TcB and TSB measurements was not recorded. The instructions were that all samples should be taken simultaneously but drawing blood from infants can sometimes be challenging and a minor time difference might have occurred without it being recorded in the protocol. However, this is unlikely to have affected the results. Lastly, previous studies have shown that skin tone can influence TcB measurements [29], but data on skin tone was not collected in this study. However, the population in our study reflects the population of newborns in Sweden, with a range of places of origin and skin tone.

## Conclusion

Our study indicates a weak correlation and poor agreement between TcB and TSB before, during, and after phototherapy on covered and exposed skin in a population of infants requiring phototherapy. This finding applies to both home- and hospital phototherapy settings. Further, our data found low agreement of TcB with TSB levels when TBS ≥ 250 μmol/L. Based on these results, we conclude that TcB does not adequately agree with TSB in our cohort of infants requiring phototherapy, likely because these infants have higher bilirubin levels than the population typically screened for hyperbilirubinemia. It is important to be aware of the limitations of TcB measurement in managing newborns with hyperbilirubinemia, ensuring that infants with high bilirubin levels receive appropriate treatment.

## Supporting information

S1 Data Bland-Altman plots showing the agreement between total serum bilirubin (TSB) and transcutaneous bilirubin (TcB) before, during, and after phototherapy. (PDF)

S2 Data Data. (XLSX)
