# Supplementary material for: Transcutaneous bilirubin in newborns before, during, and after home phototherapy—Results from a secondary analysis of a randomized controlled trial
Source: PLoS One. 2025 Mar 25;20(3):e0320067. doi: 10.1371/journal.pone.0320067 (PMC11936187; doi:10.1371/journal.pone.0320067)
Supplement: S1 Data — (PDF) [file pone.0320067.s001.pdf]

S1 Bland-Altman plots. Presents agreement between TSB and TcB before, during and after phototherapy. Bland-Altman plots are presented for all patients and for the intervention group and control group separately. The x-axis shows the mean of TSB and TcB and the y-axis shows the difference between TSB and TcB. All bilirubin values are expressed in µmol/L. The red line represents the mean difference, and the green lines represent the limit of agreement, calculated by mean difference ± SD x 1,96

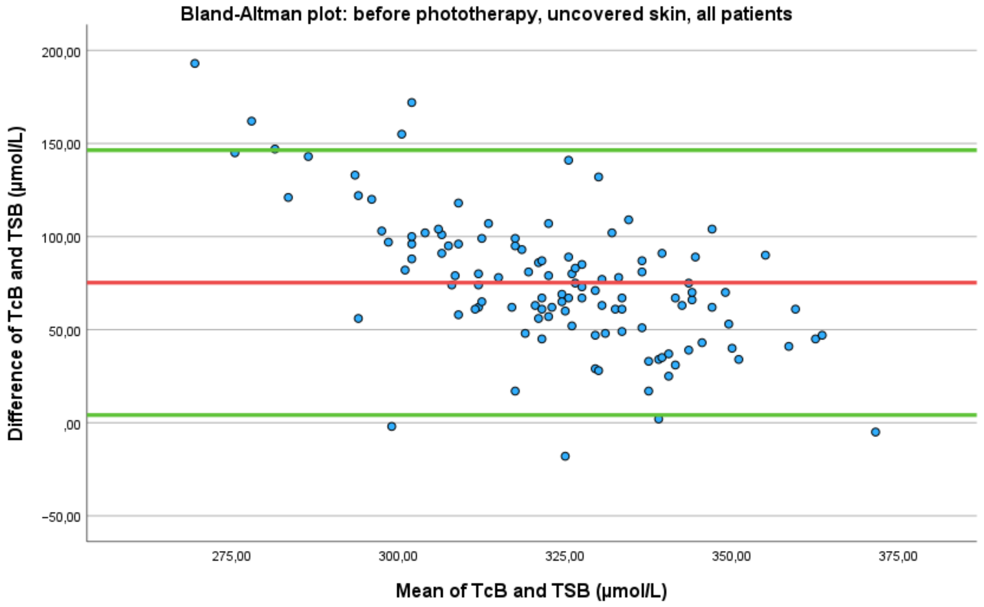

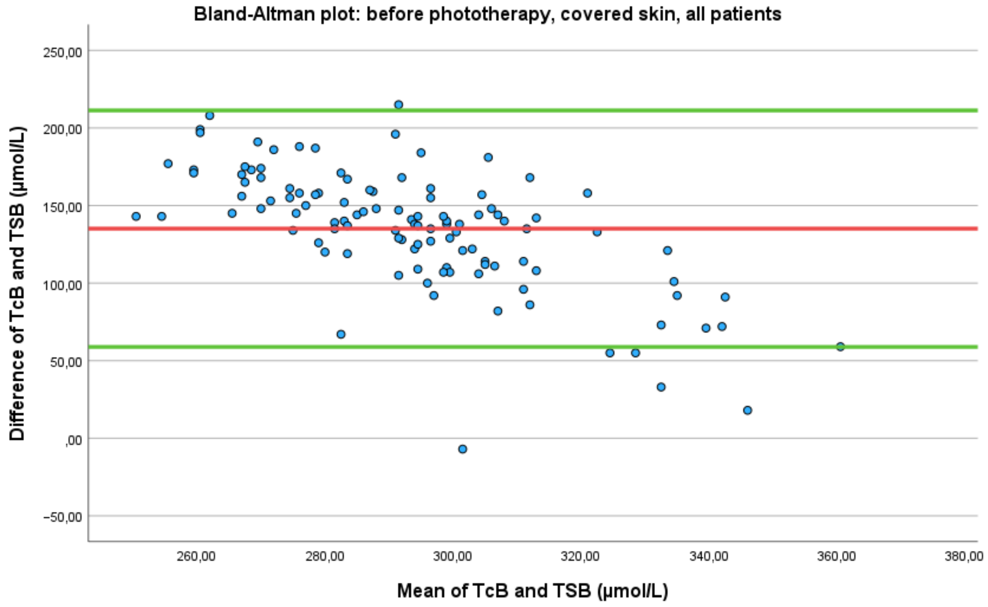

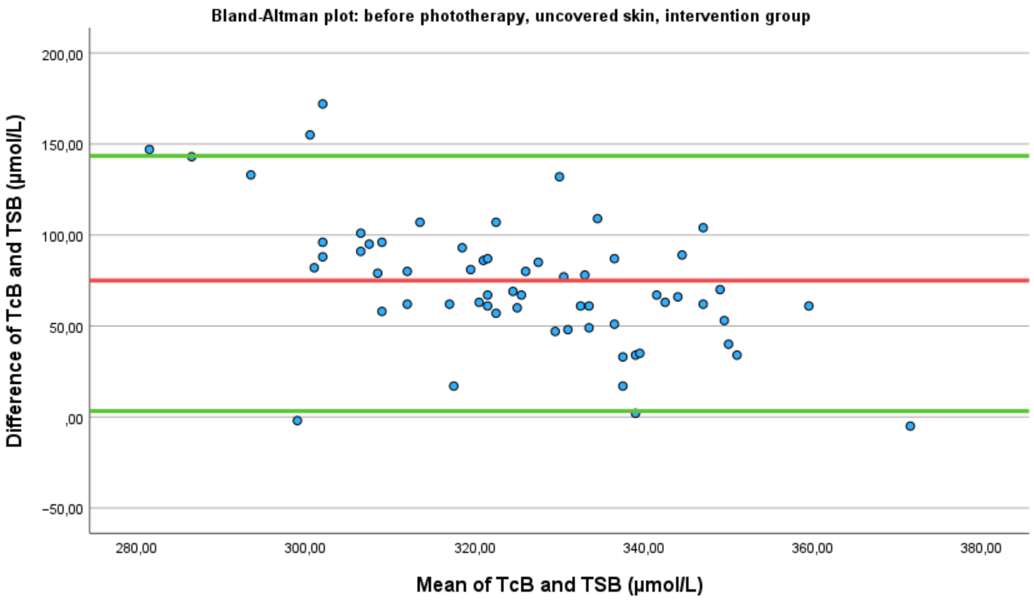
Bland-Altman plot: before phototherapy, uncovered skin, intervention group
Difference of TcB and TSB (µmol/L)
200,00
150,00
100,00
50,00
,00
-50,00
280,00
300,00
320,00
340,00
360,00
380,00
Mean of TcB and TSB (µmol/L)

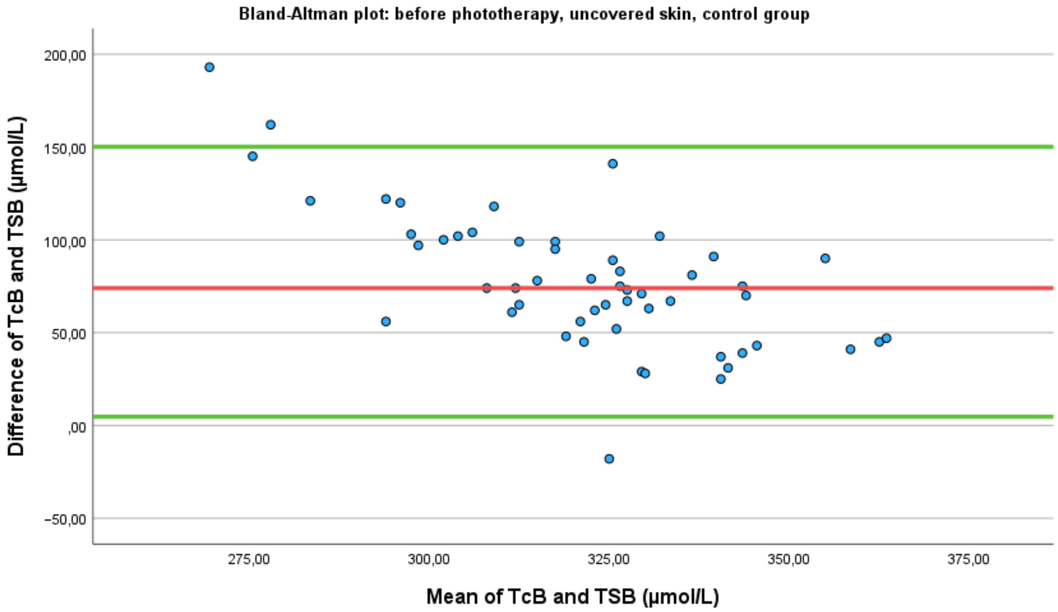
Bland-Altman plot: before phototherapy, uncovered skin, control group
Difference of TcB and TSB (µmol/L)
200,00
150,00
100,00
50,00
,00
-50,00
275,00
300,00
325,00
350,00
375,00
Mean of TcB and TSB (µmol/L)

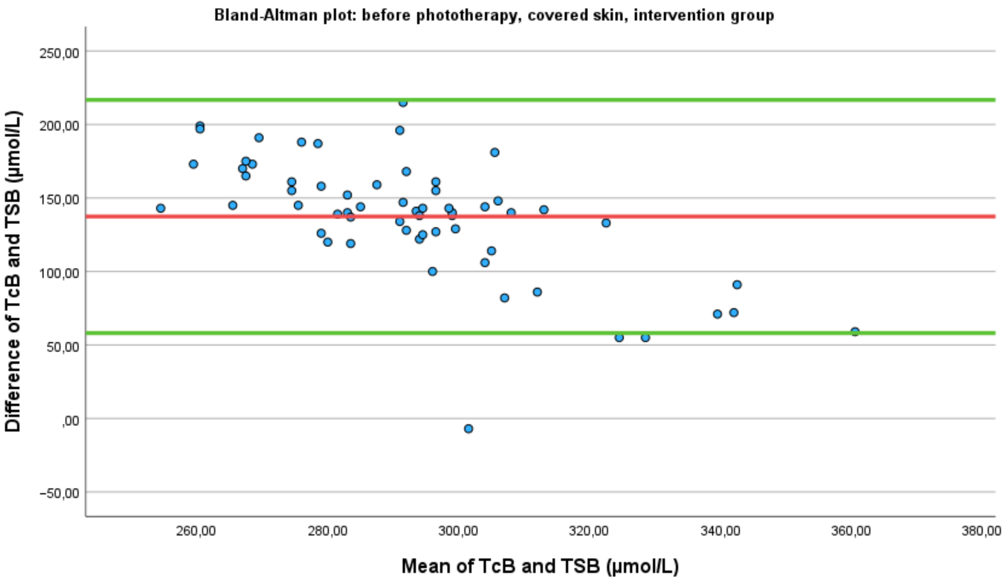
Bland-Altman plot: before phototherapy, covered skin, intervention group
Difference of TcB and TSB (µmol/L)
250,00
200,00
150,00
100,00
50,00
,00
-50,00
260,00
280,00
300,00
320,00
340,00
360,00
380,00
Mean of TcB and TSB (µmol/L)

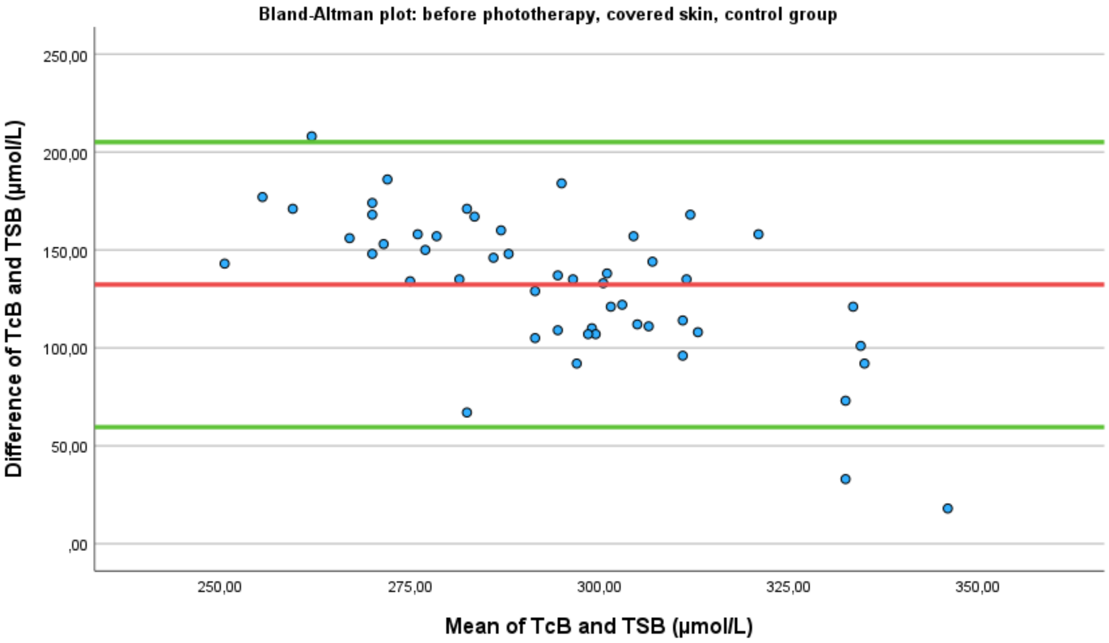
Bland-Altman plot: before phototherapy, covered skin, control group
Difference of TcB and TSB (µmol/L)
250,00
200,00
150,00
100,00
50,00
,00
250,00
275,00
300,00
325,00
350,00
Mean of TcB and TSB (µmol/L)

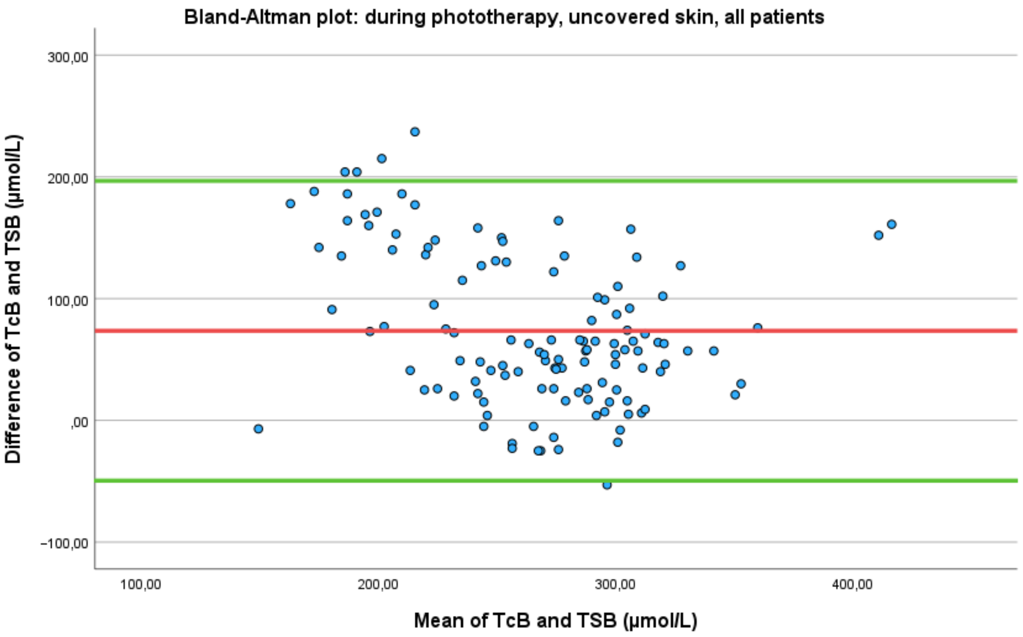
Bland-Altman plot: during phototherapy, uncovered skin, all patients
Difference of TcB and TSB (µmol/L)
Mean of TcB and TSB (µmol/L)
300,00
200,00
100,00
,00
-100,00
100,00
200,00
300,00
400,00

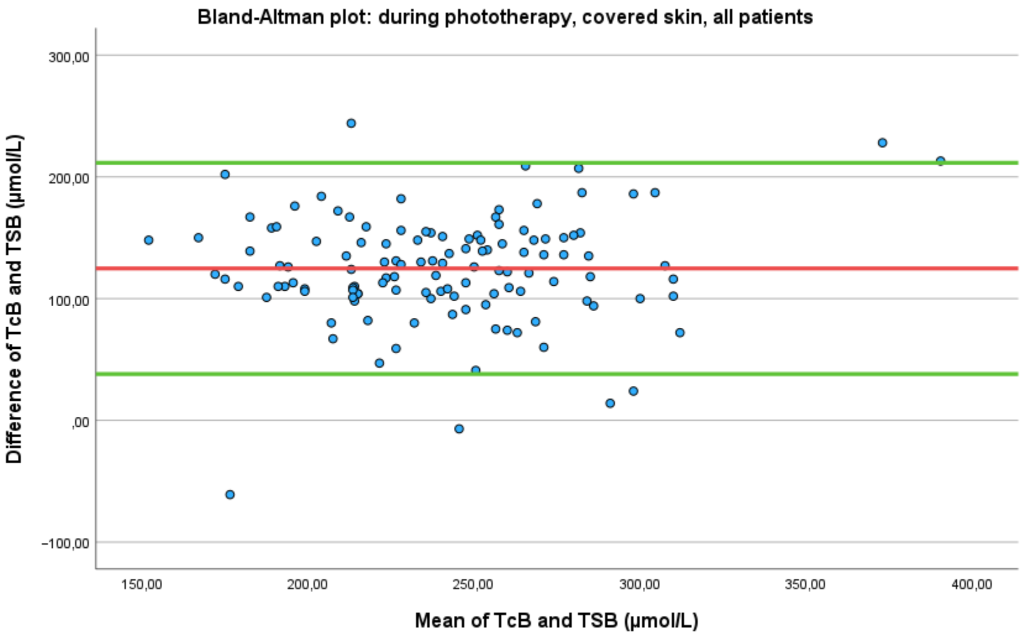
Bland-Altman plot: during phototherapy, covered skin, all patients
Difference of TcB and TSB (µmol/L)
Mean of TcB and TSB (µmol/L)
300,00
200,00
100,00
,00
-100,00
150,00
200,00
250,00
300,00
350,00
400,00

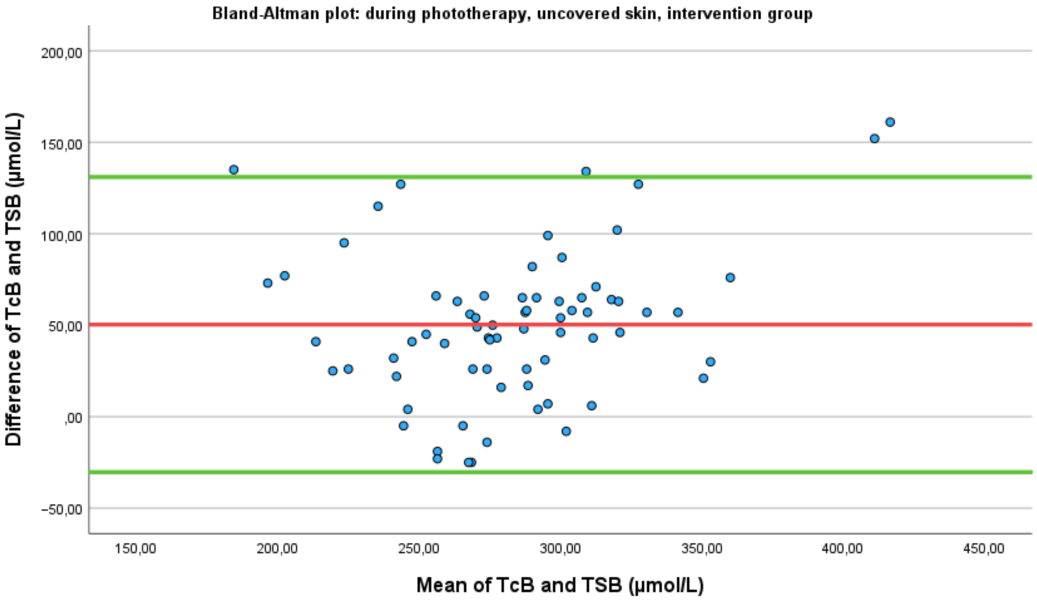
Bland-Altman plot: during phototherapy, uncovered skin, intervention group
Difference of TcB and TSB (µmol/L)
Mean of TcB and TSB (µmol/L)
200,00
150,00
100,00
50,00
,00
-50,00
150,00
200,00
250,00
300,00
350,00
400,00
450,00

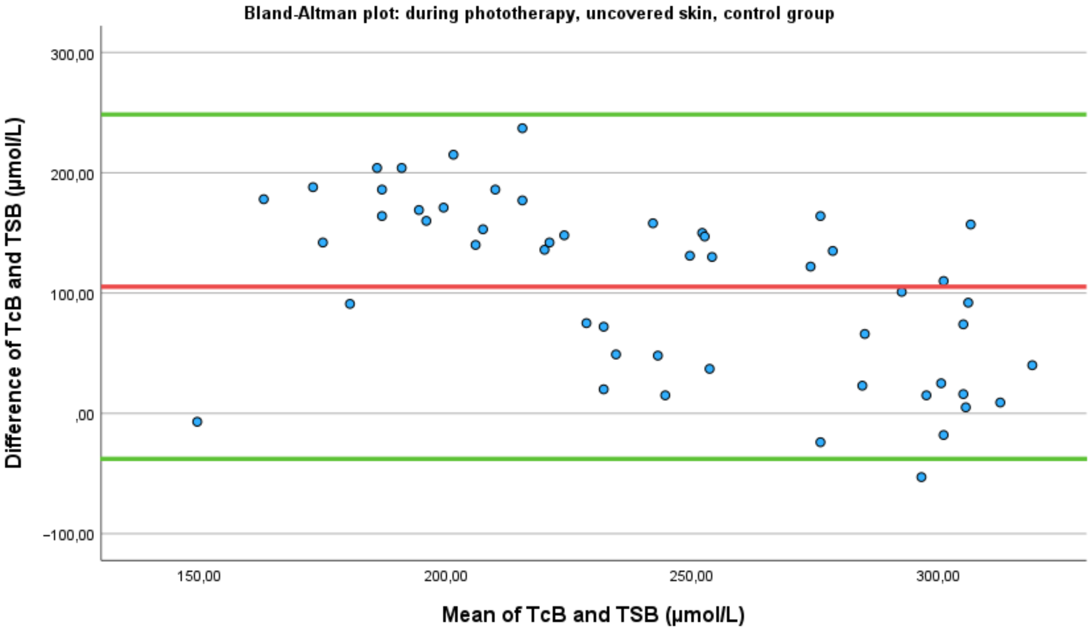
Bland-Altman plot: during phototherapy, uncovered skin, control group
Difference of TcB and TSB (µmol/L)
Mean of TcB and TSB (µmol/L)
300,00
200,00
100,00
,00
-100,00
150,00
200,00
250,00
300,00

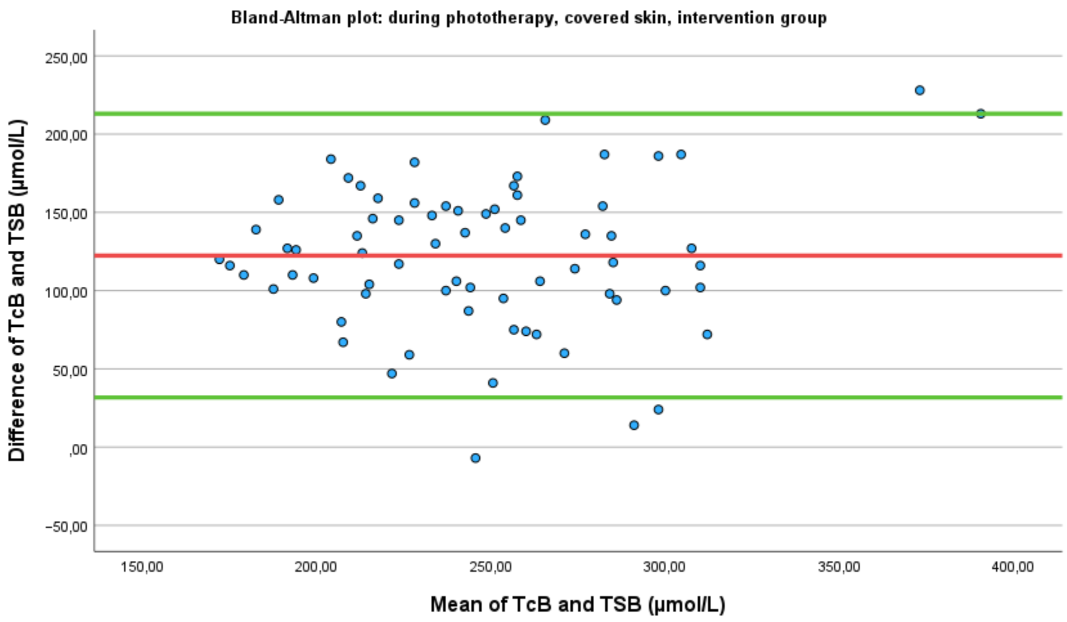
Bland-Altman plot: during phototherapy, covered skin, intervention group
Difference of TcB and TSB (µmol/L)
250,00
200,00
150,00
100,00
50,00
,00
−50,00
150,00
200,00
250,00
300,00
350,00
400,00
Mean of TcB and TSB (µmol/L)

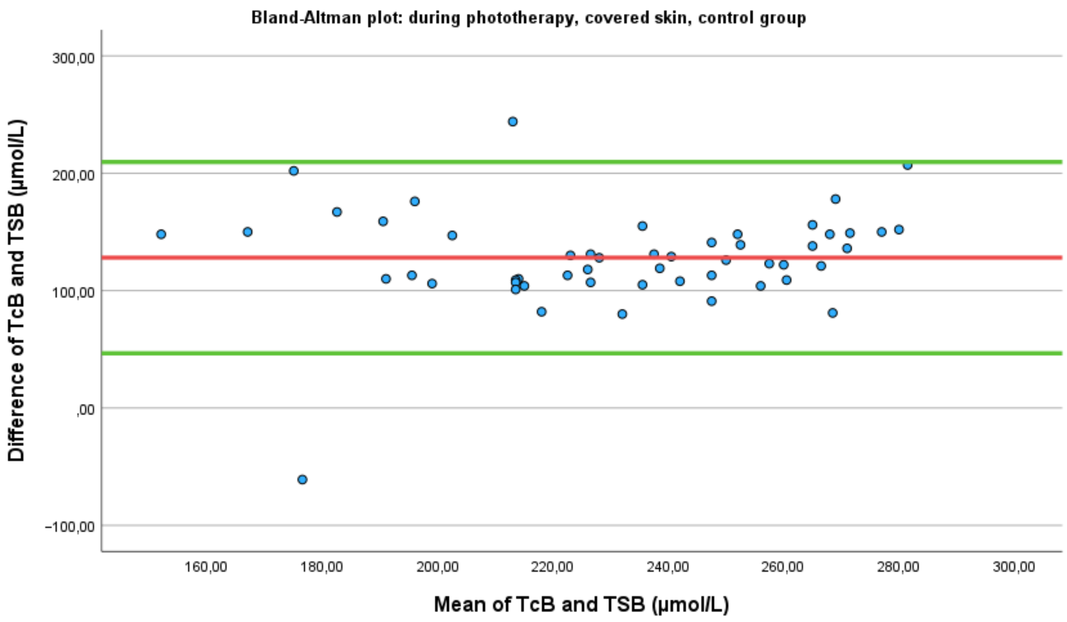
Bland-Altman plot: during phototherapy, covered skin, control group
Difference of TcB and TSB (µmol/L)
300,00
200,00
100,00
,00
−100,00
160,00
180,00
200,00
220,00
240,00
260,00
280,00
300,00
Mean of TcB and TSB (µmol/L)

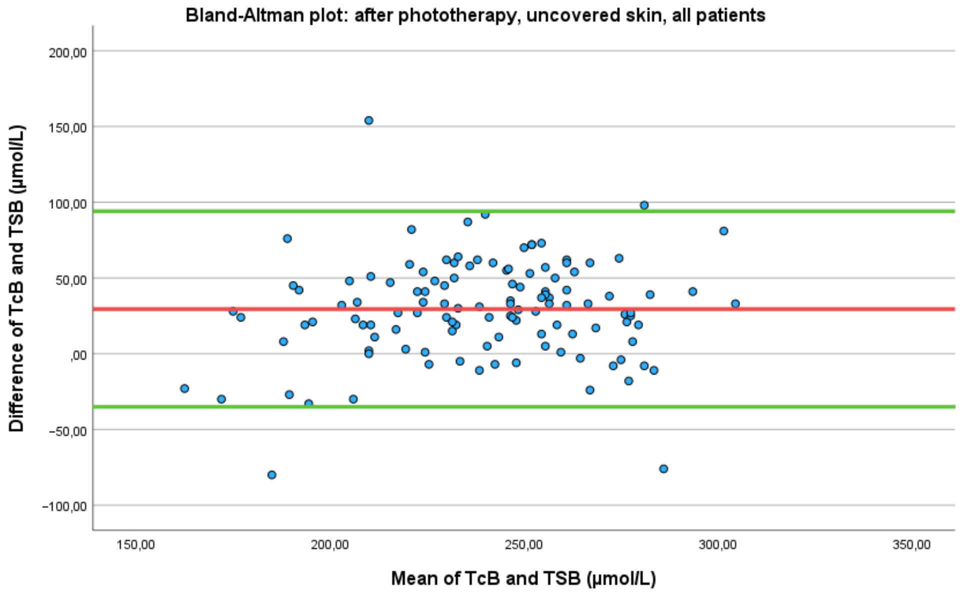
Bland-Altman plot: after phototherapy, uncovered skin, all patients
Difference of TcB and TSB (µmol/L)
200,00
150,00
100,00
50,00
,00
−50,00
−100,00
150,00
200,00
250,00
300,00
350,00
Mean of TcB and TSB (µmol/L)

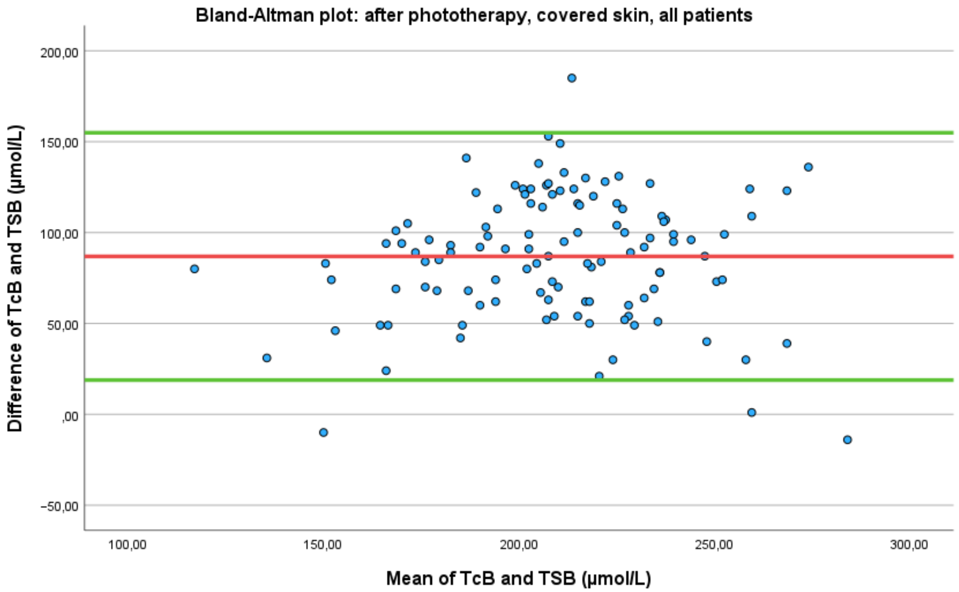
Bland-Altman plot: after phototherapy, covered skin, all patients
Difference of TcB and TSB (µmol/L)
200,00
150,00
100,00
50,00
,00
−50,00
100,00
150,00
200,00
250,00
300,00
Mean of TcB and TSB (µmol/L)

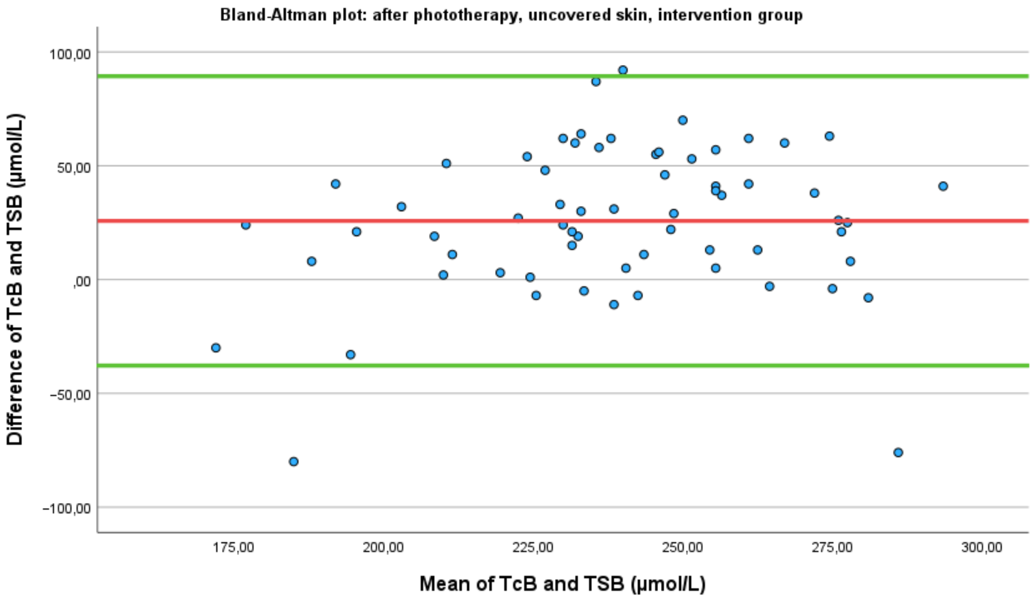
Bland-Altman plot: after phototherapy, uncovered skin, intervention group
Difference of TcB and TSB (µmol/L)
100,00
50,00
,00
-50,00
-100,00
175,00
200,00
225,00
250,00
275,00
300,00
Mean of TcB and TSB (µmol/L)

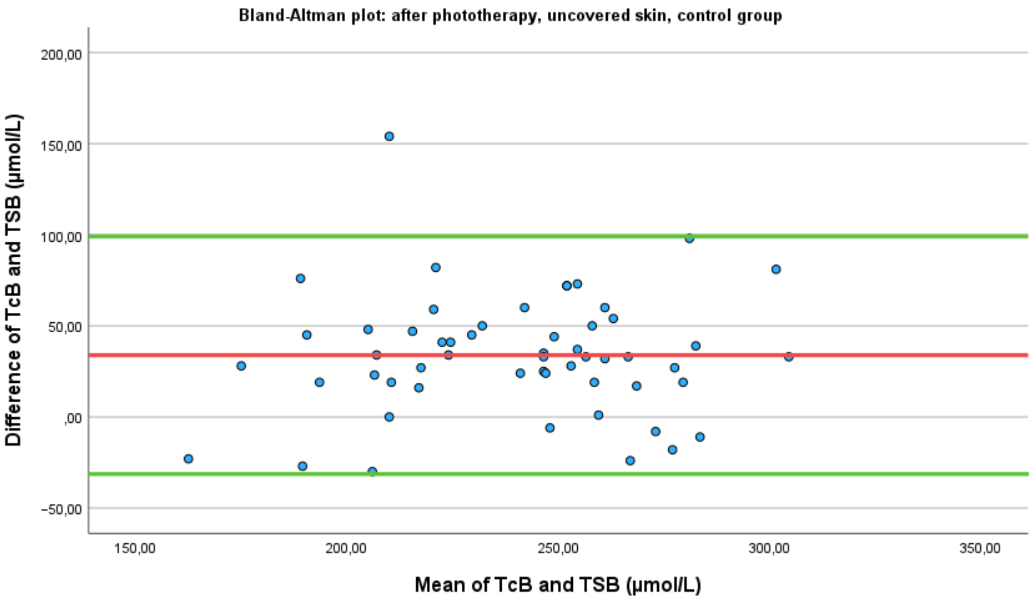
Bland-Altman plot: after phototherapy, uncovered skin, control group
Difference of TcB and TSB (µmol/L)
200,00
150,00
100,00
50,00
,00
-50,00
150,00
200,00
250,00
300,00
350,00
Mean of TcB and TSB (µmol/L)

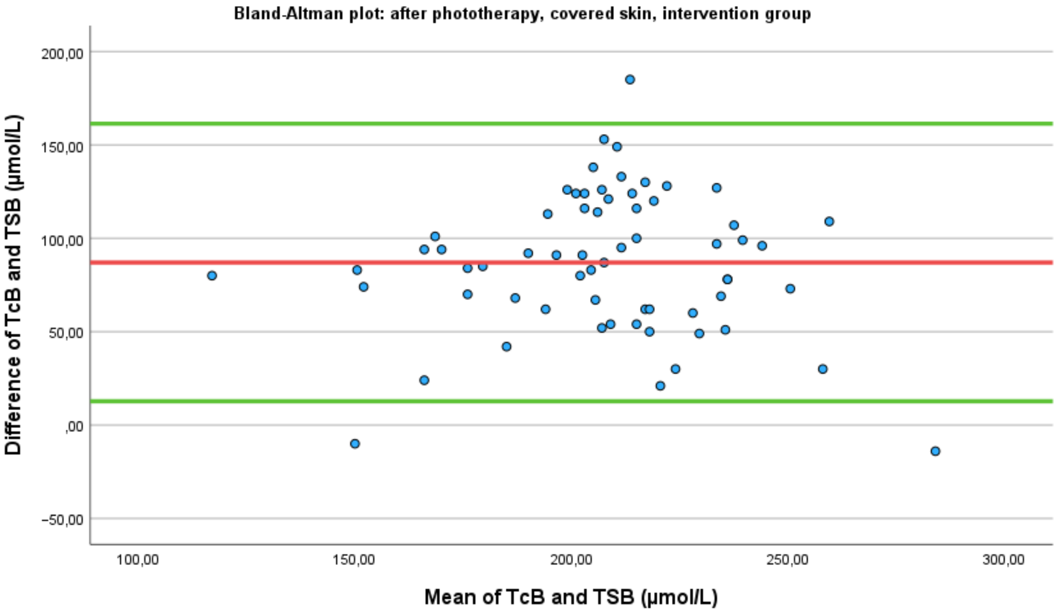
Bland-Altman plot: after phototherapy, covered skin, intervention group
Difference of TcB and TSB (µmol/L)
200,00
150,00
100,00
50,00
,00
-50,00
100,00
150,00
200,00
250,00
300,00
Mean of TcB and TSB (µmol/L)

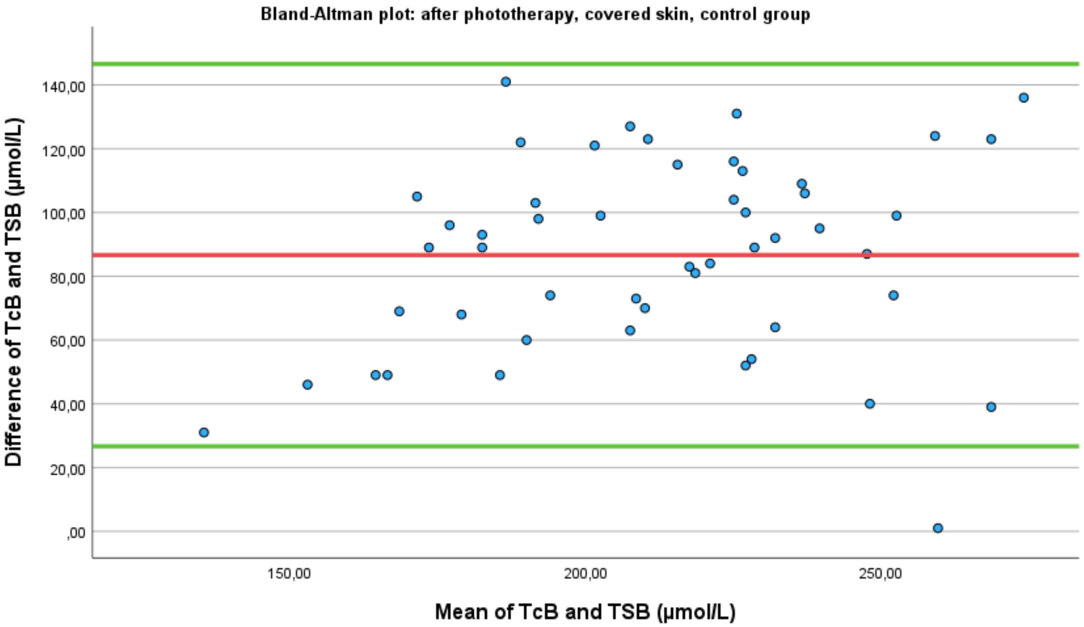
Bland-Altman plot: after phototherapy, covered skin, control group
Difference of TcB and TSB (µmol/L)
140,00
120,00
100,00
80,00
60,00
40,00
20,00
,00
150,00
200,00
250,00
Mean of TcB and TSB (µmol/L)
